# Health-related quality of life in intensive care survivors: Associations with social support, comorbidity, and pain interference

**DOI:** 10.1371/journal.pone.0199656

**Published:** 2018-06-25

**Authors:** Anne Kathrine Langerud, Tone Rustøen, Milada Cvancarova Småstuen, Ulf Kongsgaard, Audun Stubhaug

**Affiliations:** 1 Department of Research and Development, Division of Emergencies and Critical Care, Oslo University Hospital, Oslo, Norway; 2 Department of Post-operative and Critical Care, Division of Emergencies and Critical Care Oslo University Hospital, Rikshospitalet, Oslo, Norway; 3 Institute of Clinical Medicine, Faculty of Medicine, University of Oslo, Oslo, Norway; 4 Institute of Health and Society, Department of Nursing science, Faculty of Medicine, University of Oslo, Oslo, Norway; 5 Faculty of Health Science, Oslo Metropolitan University, Oslo, Norway; 6 Department of Anesthesiology, Division of Emergencies and Critical Care, Oslo University Hospital, Radiumhospitalet, Oslo, Norway; 7 Department of Pain Management and Research, Division of Emergencies and Critical Care, Oslo University Hospital, Oslo, Norway; Foundation IRCCS Neurological Institute C. Besta, ITALY

## Abstract

**Background:**

Experiences during a stay in the intensive care unit (ICU), including pain, delirium, physical deterioration, and the critical illness itself, may all influence survivors’ health-related quality of life (HRQOL). However, few studies have examined the influence of social support, comorbidity, and pain interference on ICU survivors’ HRQOL.

**Objectives:**

To investigate possible associations between social support, number of comorbidities, and pain interference on HRQOL in ICU survivors.

**Methods:**

ICU survivors responded to a survey 3 months (n = 118) and 1 year (n = 89) after ICU discharge. HRQOL was measured using the Short Form Health Survey-12 (v1), social support using the revised Social Provision Scale, pain interference using the Brief Pain Inventory–Short Form, and comorbidities using the Self-Administered Comorbidity Questionnaire.

**Results:**

Physical and mental HRQOL were reduced at both 3 months and 1 year in ICU survivors compared with the general population. This reduction was more pronounced at 3 months for physical HRQOL, while a small reduction in mental HRQOL was not clinically relevant. Social support was statistical significantly positively associated with mental HRQOL at 3 months, while number of comorbidities was statistical significantly associated with a reduction in physical HRQOL at 3 months and 1 year and mental HRQOL at 1 year. Lastly pain interference was significantly associated with a reduction in physical HRQOL at 3 months and 1 year.

**Conclusions:**

ICU survivors primarily report reduced physical HRQOL. Social support was positively associated with mental HRQOL, while number of comorbidities, and pain interference were all significantly associated with a reduction in HRQOL. Pain interference was associated with the largest reduction in HRQOL.

## Introduction

Today, most intensive care unit (ICU) patients survive their ICU stay [1], although they may experience critical illness, trauma, or deterioration of chronic disease, and many require support with a ventilator, vasoactive drugs, or dialysis. During their ICU stay, patients may experience sleep deprivation [2–4], pain [5], discomfort [6], delirium [5], and physical deterioration [7]. All of these experiences, and the critical illness itself, may negatively be associated with ICU survivors’ health-related quality of life (HRQOL) long after their ICU stay [8–16]. Previous research on this population has demonstrated that preexisting disease or comorbidity has a negative impact on HRQOL following an ICU stay [8, 17, 18], and that chronic pain had a negative impact on HRQOL [19]. Quality of life (QOL) is a multidimensional concept that, in its broadest interpretation, comprises almost every aspect of life, includes numerous definitions [20, 21]. For the purposes of this study, we used the definition of QOL that regards a person’s sense of satisfaction or dissatisfaction with areas of life that are important to them [22]. HRQOL, then, is QOL in the context of health and illness [23].

Research on patients with cardiac diseases have shown that social support may predict better health and better physical function [24]. Cancer research has shown that help from friends and family is important for patient recovery and coping [25]. ICU survivors often undergo dramatic changes in health and functioning [26–28]. Their social life may also be altered because of reduced contact with family and friends during their ICU and hospital stays, and because they are absent from work, school, and/or leisure activities. It may take a long time for these patients to regain normal activity levels, if they ever do [29, 30]. We hypothesized that these factors could explain changes in HRQOL. Previous research [31] found that instrumental and emotional social support influence HRQOL in ICU survivors; however, to our knowledge, little other research has been conducted on social support in ICU survivors and its impact on HRQOL. The aim of the present study was to investigate a possible association between social support, pain interference, and comorbidity on HRQOL 3 months and 1 year after ICU discharge.

## Material and methods

This was an exploratory study with a longitudinal design and two data collection time points: 3 months and 1 year following ICU discharge.

### Settings and sample

ICU survivors from two mixed surgical and medical ICUs (ICU 1 and ICU 2) at Oslo University Hospital, a tertiary referral hospital, were included. ICU 1 and ICU 2 have 11 and 9 beds, respectively, and neither treats trauma patients. The study took place from May 2010 to January 2014. ICU survivors aged 18 years or older with an ICU stay longer than 48 hours were invited to participate. The other inclusion criterion was the ability to read, write, and understand spoken Norwegian, so that the participant could complete the study questionnaires; patients with reduced cognitive function or terminal illness were excluded. Level of cognitive function and/or terminally ill status was established with assistance from the ICU survivor’s next of kin if they were unable to speak with the investigator (AKL) over the phone. Of the 348 patients contacted, 193 consented to participate. Among these, 118 and 89 patients completed the questionnaires at 3 months and at 1 year, respectively.

### Data collection

Three months after discharge, all eligible patients were contacted by telephone, informed about the study, and invited to participate. The ICU survivors who consented to participate got the questionnaire, study information in writing and informed consent by mail. If the ICU survivors did not reply within 14 days they got the questionnaire, information and consent form once more. The first author (AKL) used ICU electronic medical records to collect information on diagnosis, length of ICU stay, days on ventilation, and disease severity. Study participants completed questionnaires regarding HRQOL, social support, and pain interference; they also reported demographics (i.e., age, sex, education, marital status, and children), and comorbidities.

#### Severity of disease during intensive care unit stay

Two measures were used to assess disease severity during ICU stay: the Simplified Acute Physiology Score II (SAPS II) and the Sequential Organ Failure Assessment (SOFA) score. The SAPS II was developed to quantify the likelihood of hospital mortality in ICU patients [32] and is based on multiple parameter values (vital signs, Glasgow Coma Scale, and the presence of malignancy or human immunodeficiency virus infection) measured during the first 24 hours after ICU admission. These values are summed for a total SAPS II (range 0–163), with a higher score indicating greater severity of illness. SAPS II has been shown valid and reliable for use in medical, surgical [32], and coronary ICU patients [33]. A SAPS II score of 29 indicate a mortality of 10% and if the SAPS II score increase to 40 the morality also increases to 25%. If the SAPS II score increase to 64 and above the morality increase to 75% [32].The SOFA score describes different levels of organ failure over time, as well as the risk of death from sepsis, and has been used with many critically ill patients and patient groups [34]. The SOFA score has been shown valid and reliable in adult ICU patients [35]. The SOFA score is based on respiratory, cardiovascular, hepatic, coagulation, renal, and cognitive failure, each rated on a 4-point scale (total score range 0–24). A higher SOFA score indicates a higher level of organ failure and a higher risk of death. SOFA score of 0–6 may on a group level indicate mortality of 10%. If the SOFA score increase to 7–9 mortality increases to 15–20%, and SOFA score of above 15 indicates mortality of above 90% [35]. Both SAPS II and SOFA scores provide information about critical illness severity and can be used to predict mortality.

#### Comorbidities

Comorbidities were assessed using the Self-Administered Comorbidity Questionnaire (SCQ) [36], which includes 13 common and three optional medical conditions. The SCQ allows the informant to report both severity of comorbidities and their impacts on their daily life. For this study, four common comorbidities (headache, skin diseases, bowel diseases, and muscular diseases) were added to the standard 13. Only the total number of comorbidities was used in our analyses, but the most common comorbidities are presented.

#### Heath-related quality of life

HRQOL was scored using the Short Form Health Survey-12 (v1) (SF-12), which measures total health status. The SF-12 (v1) consists of 12 questions about eight health concepts: physical functioning, role-physical, bodily pain, general health, energy/fatigue, social functioning, role-emotional, and mental health. These concepts are summed to create a Physical Component Summary (PCS) and a Mental Component Summary (MCS) [37]. The SF-12 PCS and MCS are calculated using norm-based data from the 1998 general US population, with a mean of 50 (standard deviation [SD] 10), which is similar to the general Norwegian population, which has a mean PCS of 50.3 (SD 8.8) and mean MCS of 50.6 (SD 9.9) [38]. The mean cutoff score of 50 means that if the study sample scores a mean value for PCS below 50, then they have reduced physical health compared with the general population; if the study sample scores higher than 50, then they have better physical health compared with the general population. This also applies to the MCS. Higher summary scores indicate better HRQOL. The SF-12 is widely used and has been validated for use in many patient groups, as well as the general population, and has been translated into many languages, including Norwegian [38]. The SF-12 has specifically been shown to be valid and reliable in relation to, and to be a good alternative for, the longer SF-36 [37, 38].Based on recommendation from Ware et al [39] a reduction of 5.0 in PCS and MCS score was considered to be clinical relevant in this present study.

#### Social support

Social support was measured using the revised version of the Social Provision Scale (SPS) [40–42]. The revised SPS consists of 16 assertions about social support that may apply to the individual. Responses options include: strongly disagree, disagree, agree, and strongly agree, representing the degree to which each assertion describes the individual’s social support situation. A high score indicates a high level of social support [43]. The 16 assertions are summed to create four provisions—reassurance of worth, attachment, nurturance, and social integration—each of which has a maximum score of 16, consistent with previous research [43]. The total SPS score is calculated by summing the scores of the four provisions (maximum score 64). The revised SPS has been shown to be valid and reliable for use with older adults living in Norway [43], and the original SPS has been used in studies of hospital nurses [44], pregnant women, first time mothers [45], and schoolteachers [46]. The primary differences between the original and revised SPS are the decrease from six to four provisions, respectively, and a change in total score from a maximum of 96 in the original to 64 in the revised version. There are no cut off value to this instrument [43].

#### Pain interference

Pain was evaluated using the Brief Pain Inventory–Short Form (BPI–SF) [47, 48]. The BPI–SF assesses pain occurrence, intensity, location, relief, and interference with function. The BPI–SF has well-established validity and reliability in different patient groups [49–54]. The ICU survivors in our sample were divided into groups based on their answer to the first BPI–SF question, “Do you have pain?” The pain group included those who responded “yes”, and the no-pain group included those who responded “no” or whose scores were “0” on all four dimensions, consistent with previous use of the instrument [55, 56]. Pain interference with the seven functional domains was rated on a numerical rating scale from 0 (does not interfere) to 10 (completely interferes). In our analyses, we only used the pain interference score of the two highest interference score. We chose this, rather than the mean of all seven pain interference scores (in BPI-SF), because the latter would be too general and inadequately describe the sample.

### Ethics

The study was approved by the Hospital Data Inspectorate and the Regional Committees for Medical Research Ethics in Norway (reference number 2012/4b S-07505b). Only patients who gave informed consent participated; written consent was administered 3 months after ICU discharge. The study is also registered in Clinical Trials: NCT02279212.

### Statistical analyses

Sample characteristics are presented as the mean and standard deviation (SD), median and interquartile range (IQR), or proportions with percentages. Associations between social support, number of comorbidities, pain interference, and HRQOL were tested using a linear regression model at 3 months, and again at 1 year, after ICU discharge. For the linear regression analysis, ICU survivors in the no-pain group (based on BPI–SF) were assigned a pain interference score of 0. P-values < 0.05 were considered statistically significant, and all tests were two-sided. All analyses were performed using IBM SPSS (version 23; IBM SPSS, Armonk, NY, USA).

## Results

### Demographic- and clinical characteristics of the sample

The mean age of the sample was 55.1 years (SD 14.4), 63.6% (n = 75) were male, 62.7% (n = 74) were married or had a partner, and 77.1% (n = 91) had children (either young or adult children) (Table 1). The mean SAPS II and SOFA score was 44.9 (SD 16) and 8.8 (SD 3.4), respectively. The median number of days on a ventilator in the ICU was 6.0 (IQR 3–12), and the median ICU length of stay (LOS) was 9.0 (IQR 5–15). The mean number of comorbidities was 2.3 (SD 1.7), most common of which were back/neck pain at 30.9% (n = 30), hypertension at 29.9% (n = 29), cardiac disease at 27.6% (n = 27), headache at 20.8% (n = 20), and cancer at 15.8% (n = 15). The two interference items with the highest mean scores were “interference with normal work” and “interference with daily activity” after ICU discharge and both mean scores increased slightly compared with 1 year [57].

**Table 1:** Survivors’ demographics, clinical characteristics, and social provision scale scores 3 months after intensive care unit discharge.

Characteristics	3 months
	Mean (SD)
**Age**	55.1 (14.4)
**Number of comorbidities**	2.0 (1.6)
**Sex**	**n (%)**
Male	75 (63.6)
Female	43 (36.4)
**Education**	
Primary	59 (50.9)
Secondary	15 (12.9)
University/College	42 (36.2)
**Marital status**	
Married/partnered	74 (62.7)
Divorced/separated/widowed/unmarried	44 (37.3)
**Children**	95 (80.5)
Children younger than 15 years old	31 (26.3)
Children older than 15 years old/adult children	64 (54.2)
No children	23 (19.5)
**Significant negative life incidents** (during last 4 weeks)	
Death in family or close friend	18 (18.6)
Severe financial problems or living conditions	4 (4.2)
**Clinical characteristics**	**Median (IQR)**
ICU LOS (days)	9 (5–15)
MV duration (days)	6 (3–12)
	**Mean (SD)**
SOFA score	8.8 (3.4)
SAPS II score	44.9 (16.0)

SD = standard deviation, IQR = interquartile range, ICU LOS = Intensive care unit length of stay, MV = mechanical ventilation, SOFA = Sequential Organ Failure Assessment, SAPS II = Simplified Acute Physiology Score II

### Health-related quality of life in intensive care unit survivors at 3 months and 1 year

Overall, we found that compared with normative values (mean 50, SD 10), ICU survivors had a clinically relevant reduction in HRQOL PCS scores (mean 39.3, SD 10.9) and a minor reduction in HRQOL MCS scores (mean 47.7, SD 10.9) at 3 months. At 1 year, the mean PCS score increased to 43.4 (SD 12.0), and the mean MCS score normalized at 49.3 (SD 10.3). The improvement in PCS scores from 3 months to 1 year was statistically significant (*p* < 0.01), however the small improvement in MCS scores did not reach the level of statistical significance (Table 2).

**Table 2:** Changes in social support and health-related quality of life from 3 months to 1 year after intensive care unit discharge.

	3 months n = 118 Mean (SD)	1 year n = 89 Mean (SD)	95% CI	p-value
**Total SPS score**	56.1 (6.3)	54.0 (6.5)	[0.47; 3.63]	0.01
**Individual provisions**				
Reassurance of worth	14.7 (1.7)	14.4 (1.9)	[–0.11; 0.77]	0.13
Attachment	14.9 (1.7)	13.2 (2.5)	[1.01; 2.27]	<0.01
Nurturance	12.2 (2.7)	12.3 (2.8)	[–0.74; 0.53]	0.75
Social integration	14.3 (1.7)	14.1 (1.8)	[–0.23; 0.59]	0.38
**SF-12**				
PCS score	39.3 (10.9)	43.4 (12.0)	[–7.0; –2.2]	<0.01
MCS score	47.7 (10.9)	49.3 (10.3)	[–3.2; 1.4]	0.43

SD = standard deviation, 95% CI = 95% confidence interval, SF-12 version 1 Physical Component Summary (PCS) and Mental Component Summary (MCS) relative to 1998 US Population baseline, SPS = revised Social Provision Scale. Statistical test: paired samples t-test, SF-12 = Short Form Health Survey-12

### Influence of social support, comorbidities, and pain interference on health-related quality of life

Total social support had a statistically significant positive association with MCS at 3 months (*p* < 0.01), but not on PCS at 3 months. Total social support was not statistically significantly associated with either PCS or MCS at 1 year (Table 3). Of the individual provisions, only attachment was statistically significant negatively associated with PCS at 3 months (*p* = 0.02) and a positively associated with MCS at 1 year (*p* = 0.03) (Table 3). There was a statistically significant reduction in total SPS scores from 3 months to 1 year, and in attachment from 3 months to 1 year (Table 2).

Number of comorbidities was statistically significantly negatively associated with PCS at both time points (*p* < 0.01) and with MCS at 1 year (*p* = 0.01) (Table 3). Pain interference with both normal work and daily activity was associated with a clinically relevant and statistically significant reduction in PCS at 3 months (*p* < 0.01, each) and 1 year (p = 0.03 and p = 0.05, respectively). Only pain interference with normal work’s association with MCS was statistically significant at 3 months (*p* = 0.02) (Table 3).

**Table 3:** Influence of social support, comorbidity, and pain interference on health-related quality of life 3 months and 1 year after intensive care unit discharge.

	Physical Component Score			Mental Component Score		
Variable	B	95%CI	p-value	B	95%CI	p-value
**3 months**						
Total SPS score	-0.22	[–0.51; 0.08]	0.15	0.60	[0.32; 0.89]	<0.01
Reassurance of worth	0.06	[–1.45; 1.58]	0.93	-0.01	[–1.58; 1.56]	0.99
Attachment	–1.75	[–3.20; -0.31]	0.02	1.25	[–0.24; 2.75]	0.10
Nurturance	-0.19	[–0.97; 0.59]	0.64	0.28	[–0.53; 1.08]	0.50
Social integration	0.51	[–1.02; 2.04]	0.51	0.61	[–0.98; 2.19]	0.45
Number of comorbidities	–2.44	[–3.74; –1.14]	<0.01	–1.19	[–2.53; 0.16]	0.08
Pain int.–Daily activity	-10.92	[–14.61; –7.24]	<0.01	-3.93	[–8.22; 0.36]	0.72
Pain int.- Normal work	–12.08	[–15.61; –8.56]	<0.01	–4.89	[–9.00; –0.78]	0.02
**1 year**						
Total SPS score	0.57	[–0.47, 0.58]	0.83	0.34	[–0.14, 0.82]	0.17
Reassurance of worth	0.64	[–0.84; 2.13]	0.39	0.48	[–0.78; 1.73]	0.45
Attachment	–0.41	[–1.58; 0.76]	0.49	1.09	[0.11; 2.08]	0.03
Nurturance	0.09	[–0.85; 1.02]	0.85	–0.08	[–0.87; 0.71]	0.84
Social integration	–0.33	[–2.03; 1.36]	0.70	–0.50	[–1.92; 0.93]	0.49
Number of comorbidities	–3.25	[–4.40; –2.10]	<0.01	–1.66	[–2.63; –0.70]	0.01
Pain int. -Daily activity	–7.52	[–12.73; –2.30]	0.05	–2.48	[–7.88; 2.92]	0.36
Pain int.- Normal work	–7.39	[–12.13; –2.66]	0.03	–1.93	[–6.85; 3.00]	0.44

95% CI = 95% confidence interval, SF-12 version 1 Physical Component Summary (PCS) and Mental Component Summary (MCS) relative to 1998 US Population baseline, SPS = revised Social Provision Scale. Pain int. = pain interference. Statistical analysis: Linear regression

## Discussion

We found statistically significant and clinically relevant reduction in PCS for the ICU survivors compared with the normative population at both 3 months and 1 year after ICU discharge, but only a minor reduction in MCS. Further, there was a statistically significant reduction in total social support from 3 months to 1 year. Only one of the provisions, attachment, was statistically significantly associated with PCS at 3 months and with MCS at 1 year.

Our findings on HRQOL are consistent with previous studies over the last two decades [13–15, 21, 58–60]. The general findings from previous studies of ICU survivors have shown a large reduction in physical HRQOL after an ICU stay [15, 58–60], whereas the reduction in mental HRQOL is commonly found to be smaller. Our sample of ICU survivors’ mental health was similar to the normative population 1 year after discharge. The theory of response shifts [61] may explain our participants’ unchanged mental HRQOL reports. According to theory of response shifts [61], ICU survivors change their internal standards, values, and conceptualization of QOL—a response shift—and thus may report their MCS similar to that of the normative population.

One explanation for the finding that social support decreased from 3 months to 1 years, is that at 3 months, the ICU survivors were still in a rehabilitation situation, during which family and friends might have been aware that social support was more important, whereas at 1 year, this need for support may have normalized, and friends and family would likely have shifted their focus from the ICU survivor’s rehabilitation to normal activities, possibly leading the ICU survivor to perceive less social support and feel less attached to their social network.

It is difficult to compare our SPS and individual provision scores with other studies in which the original SPS was used, because the instruments have different maximum scores. However, if we compare our data with those of first time mothers [45], it appears that our ICU survivors perceived a higher level of total social support and scored higher on all provisions, except nurturance, compared with first time mothers. Both of these groups have undergone life-changing events, but with very different consequences. ICU survivors are in a rehabilitation situation, trying to regain their normal lives, whereas new mothers are learning new skills and adapting to a new life. This may be the reason ICU survivors score higher on social support. Even though social support influences HRQOL, and some aspects of that influence were statistically significant, it was moderate and of questionable clinical relevance (Table 3). Other researchers, including Tilburg [31], have found that social support has a positive impact on HRQOL. Our interpretation is that social support matters, but since our relatively small sample scored high on most of the individual provisions, there may have been a ceiling effect.

The ICU survivors in the present study also scored high on every SPS provision. This might be explained by the fact that most of these ICU survivors were married or had a partner, had children (young and/or adult), and were middle-aged, suggesting that they had networks of family, friends, and colleagues. The one individual provision on which they scored lower was nurturance. This might be explained by the mean age of the ICU survivors: many had adult children who did not need parental care in the same ways as when they were children. Some of the older ICU survivors may also have received nurturing from their adult children.

Number of comorbidities was statistically significantly associated with PCS at both time points and on MCS at 1 year. Orwelius et al. [17] found that preexisting disease had a significant influence on HRQOL after an ICU stay, and emphasized the importance of considering comorbidities when discussing outcomes in ICU survivors. We agree that this is an important consideration, especially when discussing physical HRQOL, since PCS seems most impacted by comorbidity. In regard of what is a clinical relevant change in HRQOL we chose to rely on Ware’s [39] suggestion from 1993 with a 5 point change in PCS or MCS. Based on this limit the number of comorbidity did not have a clinically relevant association with HRQOL, but others have argued that for an individual smaller change (1–2 points) could be of relevance [62]. The theory of response shift may be the reason here [62].

Pain interference had the largest association with HRQOL, and specifically on PCS. These findings are in accordance with previous research [19]. As mentioned above, the reduction in PCS was larger at 3 months than at 1 year. Of interest, the pain interference score increased from 3 months to 1 year, but had less association with PCS. We can only speculate that there is a change from rehabilitation and hope of returning to life as it was before the ICU stay at 3 months, to acceptance that life has changed at 1 year. This may have led to the improved HRQOL scores in our results and others, and may help to explain why those who experience pain interference feel that the pain has a larger influence on their life, but less impact on their HRQOL. This phenomenon could be due to yet another response shift [61].

Based on these findings and previous research [13, 14, 21, 58, 63], it seems that what most ICU-survivors need is rehabilitation of physical health. Previous research [59] has found that HRQOL improves even from ICU discharge to hospital discharge, and suggested that rehabilitation must start early. Another study [64] found that early mobilization in the ICU improved physical function after ICU discharge. Early mobilization in the ICU seems to be one way of improving physical HRQOL after an ICU stay, and this may be a specific topic for further research. Unfortunately, there is still no high-quality intervention to improve HRQOL in ICU survivors. This may be a subject for future research.

### Limitations

First, we did not have pre-admission baseline measurements with which to compare HRQOL and social support, number of comorbidity and pain interference because nearly all ICU admissions are the result of emergencies. Collecting any baseline data from ICU survivors is difficult, but previous studies have shown that this population has lower HRQOL than the normative population, even before their ICU stay [8, 17]. Second, we compared PCS and MCS derived from our sample with 1996 normative US data. Ideally, we would have used normative Norwegian data but, as mentioned above, Norwegian cutoff scores are similar to the US data [38]. Finally, our drop-out rate at 1 year was relatively high (32%). These factors may have resulted in a selection bias, though we did not find any differences between responders and non-responders at 1 year with regard to gender (p-value 0.71), age (p-value 0.87), ICU LOS (p-value 0.35), MV days (p-value 0.64) and SPAPS II score (p-value 0.21). More details are presented in supplementary material to Langerud et al [65]. Therefore, we assume that our results might be generalizable to other ICU survivors. Others have reported that the most severely ill are difficult to study [66, 67]; therefore, illness severity may explain our relatively high dropout rate.

## Conclusions

Physical HRQOL was reduced in ICU survivors at 3 months and 1 year compared to the normative population. These patients’ mental HRQOL was, from a clinical perspective, similar to that of the normative population. Social support was positively associated with mental HRQOL, while number of comorbidities, and pain interference were all significantly associated with a reduction in HRQOL. Pain interference was associated with the largest reduction in HRQOL.

## Supporting information

S1 Request For partisipation in research project. (DOCX)
