# Supplementary material for: Health-related quality of life in intensive care survivors: Associations with social support, comorbidity, and pain interference
Source: PLoS One. 2018 Jun 25;13(6):e0199656. doi: 10.1371/journal.pone.0199656 (PMC6016908; doi:10.1371/journal.pone.0199656)
Supplement: S1 Request — (DOCX) [file pone.0199656.s001.docx]

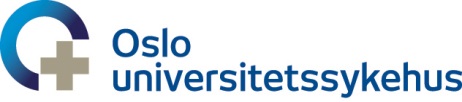


**S1 Request for participation in research project**

**Prosjekttittel:** **Project title:**

**NEVROPATI, SMERTE OG LIVSKVALITET HOS INTENSIVPASIENTER (NEUPAQ)** **NEVROPATI, PAIN AND QUALITY OF LIFE IN FORMER INTENSIVE CARE PASIENTS (NEUPAQ)**

**En studie for å kartlegge forekomst og konsekvenser av kronisk smerte hos pasienter som overlever intensivbehandling.** **A study to map the prevalence and consequences of chronic pain in patients who survive intensive care.**

I denne studien ønsker vi å vite mer om hvordan du som pasient har det etter oppholdet på intensiv. In this study we want to know more about how you as a patient have it after your stay in the intensive care unit (ICU). Hensikten er at vi som helsepersonell skal få mer kunnskap om senvirkninger av intensivbehandling som igjen kan gjøre oss bedre i stand til å yte bedre behandling/oppfølging til dere pasienter. The purpose is that we as healthcare professionals will get more knowledge about the late effects of intensive care, which in turn will enable us to provide better treatment / follow-up to your patients. Per i dag har vi ingen systematisk oppfølging av pasienter som har vært inneliggende på intensivavdelingen. As of today, we have no systematic follow-up of patients who have been admitted at the ICU. Deltakelse i studien er basert på frivillighet. Participation in the study is voluntary.

Studien går ut på at pasienter som har ligget på Generell Intensiv eller Generell Intensiv 2 The study includes former ICU patients admitted to General Intensive 1 or General Intensive 2 ved Oslo Universitetssykehus Rik shospitalet i perioden 2010-2013 svarer på noen spørreskjema 3 måneder etter utskrivelse fra Generell Intensiv / Generell Intensiv 2 ved Oslo Universitetssykehus Rikshospitalet og at de samme spørreskjemaene besvares 1 år etter utskrivelse fra Generell Intensiv / Generell Intensiv 2 . at Oslo University Hospital Rikshospitalet in the period 2010-2013. The study is a mailed survey consisting of questionnaires and a pain diary. We would like you to answer this survey at 3 months and 1 year after discharge from General Intensive / General Intensive 2 at Oslo University Hospital Rikshospitalet. Spørreskjemaene tar ca 30-45 minutter å fylle ut og spørsmålene omhandler din opplevelse av smerte, livskvalitet, angst, depresjon og posttraumatisk stress. The questionnaires take about 30-45 minutes to complete and the questions concern your experience of pain, quality of life, anxiety, depression, sleep disorders, fatigue and posttraumatic stress. The pain diary should be completed everyI tillegg følger det med en smertedagbok som fylles ut hver kveld night i en uke . for one week. Dette tar maks 5 min hver kveld. The pain diary asks questions about the prevalence of pain each day, the pains intensity and if you use any pain killers. This takes a maximum of 5 minutes each evening.

Vi ber også om tillatelse til å inn hente informasjon om ditt opphold på intensiv fra din journal. We also ask for permission to retrieve information about your stay in the ICU from your medical records. Vi er ute etter informasjon om hva som feilte deg, hvor lenge du lå på respirator, hva slags innstillinger respiratoren hadde, hvilke medisiner du fikk , om du fikk dialyse , om du hadde infeksjoner osv. We are interested in information about your diagnosis, how long you were on the ventilator, ventilator settings, medications, organ failure (e.g. dialysis), infections, etc.

**Oppbevaring av data** **Data handling and storage**

Informasjon om deg og dine svar på spørreskjemaene vil oppbevares forsvarlig innelåst og med begrenset adgang til de som gjennomfører forskningen. Information about you and your answers to the questionnaires will be kept securely locked and with limited access to those who conduct the research. Data vil bli slettet Data will be deleted or anonymized etter publisering og senest innen 31.12.2020. after publication and no later than 31.12.2020. Du har rett til informasjon om utfallet av studien og du har rett til å få innsyn i hvilke opplysninger som er registrert om deg. You are entitled to information about the outcome of the study and you have the right to gain access to what information that is registered about you. Du har rett til å få rettet eventuelle feil i opplysningene vi har registret om deg. You are entitled to correct any errors in the information we have registered about you.

Vi ber også om tillatelse til å innhente data fra din journal i ettertid hvis det skulle vise seg å være noe informasjon om din behandling som vi mangler. We also ask for permission to retrieve data from your medical records afterwards, if there should be any information about your treatment that we are missing. Alle som arbeider med studien er helsepersonell som har taushetsplikt og informasjonen vil bli behandlet konfidensielt. Everyone who works with the study is a healthcare professional who has confidentiality and the information will be treated confidentially.

I samsvar med nasjonale og internasjonale retningslinjer for forskningsetikk, vil vi med dette be om din tillatelse til å benytte da taene fra oppholdet på intensiv og svar ene dine på spørreundersøkelsen i dette forskningsprosjektet. In accordance with national and international guidelines for research ethics, we hereby request your permission to use the data from your stay in the ICU and the your answers to the survey and pain diary in this research project. Data som samles inn i denne studien vil kun brukes i tråd med studiens hensikt. Data collected in this study will only be used in accordance with the purpose of the study.

Alle lagrede opplysninger vil være avidentifiserte. All stored information will be unidentified. Ingen pasienter vil kunne gjenkjennes når studieresultatene publiseres. No patients will be able to be recognized when the results are published.

**EEtikk** **ethics**

 Det er helt frivillig å delta. It is completely voluntary to participate. Du står på ethvert tidspunkt helt fritt til å si nei til deltagelse i studien, og data lagret med tanke på denne studien vil da bli slettet. You are free to decline to the study at any time, and data stored in view of this study will be deleted. Dette vil ikke ha noen innvirkning på den fortsatte oppfølgingen og behandlingen av deg og din tilstand. This will not affect the continued follow-up and treatment of you and your condition. Du trenger ikke å oppgi noen grunn for at du vil trekke deg fra studien. You do not need to state any reason for withdrawing from the study. Dersom du ønsker å tilbakekalle samtykket, kan du kreve å få de innsamlede data slettet eller utlevert. If you wish to revoke your consent, you may require the collected data to be deleted or handed out. Dersom opplysningene allerede har inngått i vitenskapelige arbeider, har du imidlertid ikke adgang til å tilbakekalle samtykket eller kreve destruksjon av biologisk materiale, sletting eller utlevering av data (jf Bioteknologiloven §§11-14). However, if the information has already been entered into in scientific work, you do not have the right to revoke the consent or require the destruction of biological material, deletion or disclosure of data (see section 11-14 of the Biotechnology Act).

Studien har fått godkjenning fra den regionale komiteen for medisinsk forskningsetikk (REK Sør), og fra sykehusets personvernombud som ivaretar Oslo Universitetssykehus Rikshospitalet HF sitt databehandlingsansvar på vegne av Datatilsynet. The study has been approved by the Regional Committee for Medical Research Ethics (REK South), and from the hospital's local Data Inspectorate, which maintains Oslo University Hospital Rikshospitalet HF its data processing responsibility on behalf of the Norwegian Data Inspectorate.

**Ansvarlig lege for studien** **Responsible medical doctor for the study** **and principal investigator** **er seksjonsoverlege Audun Stubhaug** , (A k uttklinikken **is**

**Audun Stubhaug** (Head of the Department of pain management and research, Division of emergencies and critical care Oslo Universitetssykehus ). Oslo University Hospital).

**Spørsmål om studien kan rettes til Anne Kathrine Langerud** , intensivsykepleier ved Akuttklinikken Oslo Universitetssykehus Rikshospitalet.**Questions about the study can be directed to**

**Anne Kathrine Langerud** (Critical care nurse and PhD scholar at Department of research and development, Division of emergencies and critical care, Oslo University Hospital Rikshospitalet. Tlf 93229832 Phone +47 93229832).

 Øvrige medarbeidere er Seniorforsker Tone Rustøen (Akuttklinikkens forskingsavdeling ved Oslo Universitetssykehus ). Other employees are Senior Researcher Tone Rustøen (Department of research and development, Division of emergencies and critical care, Oslo University Hospital).

 Databehandlingsansvarlig er Oslo Universitetssykehus Rikshospitalet ved administrerende direktør. Data Processing Officer is Oslo University Hospital Rikshospitalet by the CEO.

Studien vil bli publisert i internasjonale tidskrift i form av flere vitenskaplige artikler. The study will be published in international journals in the form of several scientific papers. Disse vil inngå i flere doktorgrader (PhD). These papers will be included in a doctoral degree (PhD).

----------------------- -----------------------                                                                       ------------------------- -------------------------

Anne Kathrine Langerud Anne Kathrine Langerud                                                         Audun Stubhaug Audun Stubhaug

Intensivsykepleier/PhD stipendiat Critical care nurse / PhD scholar                                          Prof. Prof. Dr.med. MD. /Principal investigator

***Denne siden fylles ut, rives løs og sendes inn sammen med evt. spørreskjema og smertedagbok.*** ***This page should be filled out, torn off and submitted together with the questionnaire and pain diary.***

SAMTYKKEERKLÆRING for studien: CONSENT for the study:

**NEVROPATI, SMERTE OG LIVSKVALITET HOS INTENSIVPASIENTER** **NEVROPATI, PAIN AND QUALITY OF LIFE IN FORMER INTENSIVE CARE PASIENTS**

**En studie for å kartlegge forekomst og konsekvenser av kronisk smerte hos pasienter som overlever intensivbehandling.** **A study to map the prevalence and consequences of chronic pain in patients who survive intensive care.**

**Jeg bekrefter med dette å ha mottatt skriftlig og muntlig informasjon om studien, og er inneforstått med at jeg når som helst kan trekke meg fra studien uten konsekvenser for meg og behandling av min tilstand** **I hereby confirm that I have received written and oral information about the study and understand that at any time, I may withdraw from the study without consequences for me and the treatment of my condition.** **.** **Jeg er inneforstått med at deltakelse i studien er frivilllig.** **I understand that participation in the study is voluntary.** **Jeg er inneforstått med at jeg kan be om** **at innsamlet materiale slettes,** **dog ikke etter at resultatene er publisert.** **I understand that I may request** **that the collected material be deleted,** **but not after the results have been published.**

**Ja takk,** jeg ønsker å delta i NEUPAQ studien. **Yes, thank you,** I would like to participate in the NEUPAQ study.                          ……. ....... (sett kryss) (tick)

**Nei takk,** jeg ønsker ikke å delta i NEUPAQ studien **No thanks,** I do not want to participate in the NEUPAQ study               ……. ....... (sett kryss) (tick)

------------------------------------ ------------------------------------                                           --------------------------- ---------------------------

Navn (blokkbokstaver) Name (block letters)                                                         Fødselsdato date of birth

----------------------------------- -----------------------------------                                                         ---------------------------- ---------------------------- Signatur Signature                                                                                     Dato Date

 Jeg bekrefter å ha gitt skriftlig og muntlig informasjon om studien I confirm that I have provided written and oral information about the study

Studie.medarb. Study worker. ----------------------------------------------- -----------------------------------------------               D ato --------------- D ate ---------------
